# Abuse of Dominant Position, Effective Judicial Protection and Abuse of Procedure

**DOI:** 10.1017/jme.2025.10145

**Published:** 2025

**Authors:** Carmen de Vivero de Porras, Enrique Sanjuán y Muñoz

**Affiliations:** School of Law, University of Malaga, Spain

**Keywords:** competition, strategy, medicines market, dominant position, market

## Abstract

On October 21, 2022, the Spanish Competition Agency (CNMC) sanctioned the North American pharmaceutical company Merck Sharp & Dohme for abuse of a dominant position. The practice for which it was finally sanctioned consisted of the adoption of a strategy aimed at delaying and making it difficult for another company to enter the Spanish medicines market in order to protect sales for a product marketed exclusively by that company and for which had a patent. This paper analyzes this resolution in an attempt to delimit the difference between the legitimate exercise of the right to effective judicial protection and its abuse.

## Introduction

On October 21, 2022, the Spanish Competition Agency (CNMC, its acronym in Spanish) sanctioned the North American pharmaceutical company Merck Sharp & Dohme (MSD) for abuse of a dominant position.^1^ The practice for which it was finally sanctioned started in 2017 and was aimed at delaying and making it difficult for another company, Insud Pharma SL (IPH), to enter the Spanish medicines market for ring-type hormonal contraceptives in order to protect sales of its own patented contraceptive hormonal vaginal ring, “NuvaRing.” Through this strategy, MSD managed to initially prevent its competitor IPH from entering the Spanish market, and this delay caused economic losses to IPH.^2^ To do this, it used its patent rights on the NuvaRing product and generated claims, first out of court and later judicially, with the intention of delaying the entry of the second company’s novel product into the Spanish market.^3^

The strategy MSD followed was that of obstruction through judicial processes, using the exclusive rights of the patent of the dominant company. Most of the problems that arise in relation to pharmaceutical products usually develop from the assumption that the manufacturers of these patented products offer large compensation to the generic manufacturers once they sue them for obstruction.^4^ In exchange for such compensation, generic manufacturers drop their legal claims and market their products later. These are cases of “reverse payments” (Reverse Payment Settlements), since the dominant one pays to prevent others from entering the market that they dominate.

In this Spanish case, MSD did not wait for the company that intended to enter the market to sue it but rather took the initiative, intending to generate a situation similar to the one that usually occurs when competitors complain about such obstruction. The objective was to achieve the same situation that usually occurs in the North American market and possibly offer compensation in court to justify the delay. In fact, the main procedure for patent infringement, as we will see, is suspended at the request of both parties precisely because an attempt is made to reach an agreement. However, the existence of the conduct was reviewed by the CNMC and therefore any agreement became impossible.

## Evergreening Through Patent Strategies

Before analyzing the specific situation and the location of the behaviors sanctioned by the Spanish agency, it is necessary to make a brief introduction to the so-called SDiME (Strategic Delay in Market Entry) or rather to the different strategies that can be followed to limit, delay, or prevent entry into a market of a certain product in this sector.^5^

This is what is known as “evergreening” or, in the (partial) definition of Feldman (2018), “artificially extending the life of a patent or other exclusivity by obtaining additional protections to extend the monopoly period.” More than that, it is also about another series of behaviors that, protected by law, allow said extension of protection through de facto means.^6^

As correctly stated in a work by the American Medical Association (2017), these strategies can differ.^7^

### Secondary Patenting

Firstly, the so-called secondary patents (Secondary Patenting) refer to patents that cover peripheral aspects of a medicine or its use. Such patents may include different features, but they are not sufficient for the absolute novelty requirement. An example of this occurred in *Novartis AG v Union Matter. of India* (2013).^8^ Generally, the secondary patent strategy is a common approach to extend patent protection. When new patents are obtained on aspects related to the original drug, they effectively extend the exclusivity of the brand drug in the market beyond the original patents. The monopoly that is granted consequently delays the availability of generic medicines. However, other studies have highlighted that the problem is not just delay to market entry by generic drugs, but social benefit. Patents (both primary and secondary) belonging to the creators of highly successful drugs are not meant to give them a monopoly on future innovations in relation to the drug.^9^

The majority of post-blockbuster drug innovations are done by third parties, with just over a quarter (27%) done by the drug’s creator, resulting in an average of 13 secondary patents per drug. These secondary patents, therefore, become essential given the value they acquire compared to new innovations or new patents since they are intended for the behavior that we have called the perenniality of drug developers. In other words, a secondary patent limits new patents by third parties.

This would support the view that drug creators’ secondary patenting can have adverse welfare effects by extending the creator’s market exclusivity over the drug and restricting innovation.

### Restrictions on Distribution

Another issue that appears as an attempt to expand one’s business is restricting someone else’s business. That is why, taking advantage of the need for licenses or authorizations from national authorities, the way to expel or prevent entry into the market is to attack new products either because they have not passed the general regime of requirements or because they have not been approved or obtained the necessary authorization. This can become a double regime of attack, which is generally used not only because said authorization is missing, but also because the original product’s patent is said to have been infringed.

Thus, in this last case, which is the one we are going to analyze, the construction of the strategy is based on an attack both before the authorities that must authorize it and in the courts.

Furthermore, a variation of the first strategy is to convert the new product into a collective demand so that the citizens themselves are the ones who prevent its entry into the market. This can be either because there is already the original, which is seen as effectively covering the health needs of the people and relevant diseases, compared to the generic, which is not seen as sufficiently tested or otherwise insufficient due to the better characteristics of the original.

### Reverse Payment Settlements

Any of the previous assumptions we have discussed also entail an ultimate supplement (ultima ratio) to solve the problem of the original product or manufacturer, which is paying a generic manufacturer for not making, not manufacturing, or not entering the market.^10^ This strategy can be used independently as well as complementarily to the others since, ultimately, it is about avoiding generic entry into the market (or delaying it)^11^ through agreements with those who can introduce generics.

This approach can also be used to preemptively prevent research, even before it is allowed regarding the original product, including by acquiring companies or production units dedicated to it.

This approach (return for delay) was highlighted by Feldman et al. (2016)^12^ through a triple strategic approach. They described the generations of strategies used to delay the entry of generic drugs as follows:

1. In Generation 1.0 (Pay-for-Delay), “branded companies simply pay generics to delay entering the market, reaping billions of dollars of benefit.”^13^
2. Generation 2.0 “involves paying for delay through multiple side deals that camouflage the value of the payment … [with methods including] ‘boy scout clauses’—agreements to behave honorably that actually mask anticompetitive collusion.”^14^
3. Generation 3.0 “moves from collusion to obstruction.… [It] uses administrative processes, regulatory schemes, and drug modifications to prevent generics from getting to market.”^15^

The evolution of pharmaceutical companies has focused on finding solutions to two problems: on the one hand, continuing to maintain a monopoly situation and, on the other hand, preventing their actions from being investigated for anticompetitive practices.

From there, the first stage was quickly surpassed by the next where, from an economic point of view, what is done is sharing the monopolistic market, or at least some of its profits. This approach can also be seen from two perspectives since the brand company does not always assume the costs. Instead, there may be an increase in the price of the products to cover the cost of the payment, which may delay the entry of the generic into the market. However, this delay is known to be uncertain and limited in time. Therefore, the period of greater exploitation being purchased also entails increasing profits exponentially to take advantage of the exclusive market. The product price could consequently be raised again to compensate for the loss of profits in the future. However, this increase in price in relation to the marginal cost is more easily detectable by competition agencies as the means and instruments for their investigations have evolved.

The second stage is simply a crude attempt to mask the above (multiple side deals) through multiple agreements related to the industry that identified them, and even through common projects, supporting research, and multiple agreements that distributed the cost, income, and agreement.

Finally, the third stage starts by using joint, mixed, and complex strategies that add other administrative or judicial ones. Its double objective is to delay the entry in time (holdout) and finally cover the operation with the possibility of an economic agreement. Non-aggression, however, should not prevent a competition investigation, as we analyzed in the present case.^16^

## MSD v. IPH Matter – Description of the Alleged Violation that Motivates the Strategy

The problem arises as a consequence of the medical similarity between an initial vaginal contraceptive ring and its subsequent generic. From a medical point of view, NuvaRing and Ornibel are similar, since they both use the same active ingredients (etonogestrel and ethinyl estradiol), the same pharmaceutical form (vaginal ring), and the same safety and effectiveness profile. These two products administer the active ingredient in the same way through a ring that, during use, releases the active ingredient.

MSD was the owner of the first contraceptive combined hormonal vaginal ring, commercially called “NuvaRing,”^17^ protected by the European patent EP ‘815.^18^ The patent’s expiration occurred on April 9, 2018, due to the expiry of the maximum exclusive exploitation time (20 years). In this way, and until the patent’s expiration date, MSD was the only company that could manufacture and market the product in Spain with all the technical characteristics claimed in the patent.

The claims determine the scope of protection conferred by the patent or patent application. Considering the claims, the drawings, and the description in the specific case, it is important to note the storage temperature of the ring — which must be between 4ºC and 25ºC — is an essential element in the EP ‘815 patent, to maintain the steroid in a slightly oversaturated state for a period of 6 or more months. MSD denied this section, but the decision of the competition agency understood the claim itself as follows:

The present invention is based on the surprising finding that a steroid can be retained in a supersaturated state during prolonged storage (such as 6 months or longer) at a temperature between 4ºC and 25ºC, provided that the concentration of the steroid does not exceed the solubility at 25ºC excessively.

On June 9, 2017, the Spanish Patent Office authorized the “Ornibel” ring, developed by Insud Pharma, as a generic medicine (EFG) for NuvaRing. Ornibel has its own patent (LF), which is titled “Drug delivery system for use in contraception comprising a core and a shell.” According to the information in the technical sheet, the Ornibel does not require any special storage temperature, so it can be stored for long periods without maintenance, which avoids significant expenses and costs.

From a technical point of view, the products are different, since they present essential differences in components, and fundamentally in terms of the type of polymer used and the degree of saturation of etonogestrel in the core of the polymer. According to the NuvaRing patent, etonogestrel is present in the ring core at a relatively low degree of supersaturation but is supersaturated. In the LF Ring, however, it is undersaturated. The supersaturated character of etonogestrel in the polymer is one of the claims of the EP ‘815 patent. This means that only a product that presents the same level of saturation as described in said patent will be considered an infringement of the patent.^19^

## Previous Contacts for the Final Strategy

In accordance with the resolution of the Spanish Competition Agency, the handwritten notes obtained in the investigation prove that, in March 2013, MSD was already attentive to the entry of an important competitor and paying attention to the characteristics of the new product that it was introducing, with respect to which MSD was marketing certain technical advantages at that time.

Likewise, under said investigation, the LF patent was published on August 22, 2013, in the International Patent System of the World Intellectual Property Organization. Although this is not enough to protect the contradictory patent, it is sufficient that the brochure describes the cold conservation of the patent and its non-saturation. In this way, the first claim of the MSD patent is not infringed because there will be no oversaturation, as can be seen in said document. Furthermore, this document was used by MSD both for the precautionary measure procedure and the main infringement procedure in such a way that it could not deny its validity and simultaneously affirm it according to its convenience.

But in 2014, Insud Pharma informed MSD of its intention to market its ring, for which it tried to reach an agreement with MSD to collaborate on marketing in the United States. They contacted MSD; they signed a confidentiality agreement based on non-bioequivalence and the fact that they were different products. Specifically, and to reach a licensing agreement between both companies, Insud Pharma made a presentation of its ring to MSD in which both the characteristics and degree of market penetration of NuvaRing, as well as the differences with the ring developed by Insud Pharma, highlighted that it did not infringe the NuvaRing patent, as it had different characteristics, as stated in the slides of said presentation. From that moment on, MSD knew that the product had a different polymer composition and would not be codified similarly in the USA.

As a summary of this, we have the following:

**Figure 1:**
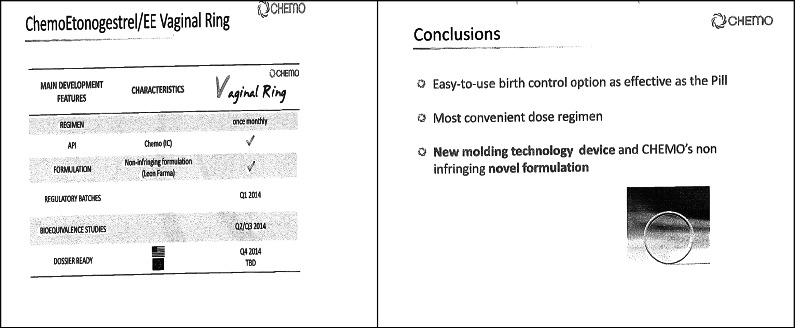
Source: CNMC Resolution

Although MSD argues that it was a different product, a licensing agreement was finally reached between both companies regarding the non-infringing (patent) formula of the ring because MSD saw not only the opportunity to relaunch its product but also to avoid generic competition. This is expressed in the final email from Insud Pharma of January 30, 2015, which has the following content:

I would like to summarize some points of the follow-up … we have developed a non-infringing formula that could be interesting for Merck, allowing it to relaunch its product and thus avoid competition from generics….

Notwithstanding the above, on May 4, 2015, MSD informed Insud Pharma of its decision to focus on a new version of NuvaRing and not continue with the collaboration proposal between both companies. With this communication, the negotiations ended.

With this, however, MSD’s actions do not end as there is (from January 2015 to June 2017) intense monitoring of the competing vaginal ring product and the development of a commercial strategy with the knowledge of IPH product features.^20^

This is derived from a document collected during the CNMC’s inspection of MSD, in which there is no mention of an alleged infringement of MSD’s patent by the Insud Pharma ring or any of the generics.

## The Facts

### Pre-Constituted Evidence in Spain

On June 13, 2017, MSD sent a notice to Insud Pharma, stating that the Ornibel ring infringed NuvaRing’s patent, and they were requested to confirm to MSD, within seven days and in writing, that all group companies would stop manufacturing, offering, importing, and marketing said ring before April 9, 2018. In the event of a negative response, it required that, within seven days, five samples of the Insud Pharma product be delivered to MSD and the additional documentary information they requested in an annex to the request letter. They warned that the lack of response or their refusal to respond would be understood as a decision to market a product that infringed MSD’s patents, which they would inform their clients so that they could take the appropriate legal measures.

On June 22, 2017, Insud Pharma, by email, responded to the request, pointing out that the Ornibel ring did not infringe any of the patents cited by MSD and conditioned the delivery of the requested samples to the prior signing of a confidentiality agreement between the parties. This desire was reiterated twice in August. The second time, MSD were even sent a confidentiality document to be signed. The proposed NDA by Insud Pharma included the following clause:

Recipient commits not to use the Product Information: 1.6.1. to file any patent infringement action (either in Spain or any other country where the Patents have been validated) against Exeltis, León Farma, any of their Affiliates, licensees or the customers of those parties; or 1.6.2. to file or amend any existing patent application (including divisionals or continuations thereof).

On September 8 and 19, 2017, Insud Pharma reiterated to MSD that they would send information required by MSD regarding its ring if MSD signed a confidentiality agreement. At that time, the ring was already sold in pharmacies.

On September 20, Insud Pharma learned of the petition presented on June 27, 2017 by MSD before the Commercial Court. It was a request for fact-checking proceedings against Insud Pharma and another requesting judicial assistance to verify the technical characteristics and manufacturing process of the Ornibel ring. That is to say, on June 13, it had been required to stop manufacturing, and on June 27, the request to verify the facts was submitted. When referring to the request in its letter, instead of stating that Insud Pharma had responded by offering the information conditional on signing an NDA, MSD said that Insud Pharma did not want to make any documentation available. The fact-checking procedures were agreed by the Commercial Court by resolution of July 14, 2017.

Notwithstanding the above, after the request for fact-checking procedures and once these had been agreed by the Commercial Court on July 14, 2017, but of which Insud Pharma would be aware only on September 20, on August 8, 2017, MSD sent another email to IPH to see if it would be interested in commenting on the development of its contraceptive ring and the possibility of finding an amicable route for the product.

Meanwhile, during the verification procedure, MSD attempted to appear in the next proceeding to be examined by the appointed expert. The court refused to permit it to appear.

However, the appointed expert did not consult Insud Pharma at any time even though she had been ordered to do so. On September 11, 2017, two days before the expert’s report was available, MSD sought precautionary measures (preliminary injunction) before the Commercial Court, relying on its own report.

The Commercial Court issued Resolution No. 203/2017 on September 18, 2017, agreeing to the precautionary measures under which the manufacture and marketing of the LF Ring (Ornibel) in Spain were prohibited. IFH was notified of these measures on September 20. A written opposition was filed against them on October 28. The hearing for the final decision was to be held on November 15 and 16, 2017.

After these ex parte preliminary injunction measures, MSD filed a lawsuit on October 26, 2017 for patent infringement. If this type of precautionary measure is obtained from a court, a lawsuit must be filed within 20 days under the sanction of annulling them. In the statement of claim, MSD also requested compensation for the damages suffered due to the alleged infringement of the patent, the final determination of which was not specified in the lawsuit, being deferred until the time of the execution of the judgment that resolves the dispute.

Thus, the precautionary measures, the lawsuit for patent infringement, and the claims for damages would run parallel from then on. IFH’s opposition to the precautionary measures is the first issue it had to present before the Courts.

After the hearing on precautionary measures on December 12, 2017, they were revoked (overruled) by Order of the Commercial Court. The Order of the Commercial Court categorically stated the following:

… it is quite artificial to request fact-checking proceedings in June; and before the deadline (September 22) for the judicial expert to issue her report – and not knowing her conclusions – the plaintiff requests precautionary measures (September 11) accompanied by an expert report of the same dates and which is later expanded with another dated October 26; and where, in both reports, the basis for the conclusion regarding the disputed fact are documents that were already available previously, that is, in June 2017….

The judge himself asked in said resolution whether or not MSD knew of the expert’s conclusions before they were presented, which is also analyzed in the resolution:

3.14 In summary, with all of the above, we see ample evidence to conclude that the expert … rather than a judicial expert, has acted as a party expert; its report being at least limited or incomplete, if not partial; taking into consideration only what benefits the plaintiffs and not investigating with zeal and curiosity what could benefit the defendants; ignoring what could harm the former and leaning, without conclusive evidence, towards what harms the latter. In this sense, we must remember the obligation established in art. 335.2 of the LEC for every expert and which, if possible, is especially required for the one in whom the court places its trust as a judicial expert:….^21^

Although MSD appealed the resolution before the Provincial Court (Court of Appeal), it was dismissed on November 13, 2018. Also, here the Court of Appeal said the following:

In any case, we do not believe that it is true that the defendants have refused to provide the polymer so that the analyses required to verify the discussed characteristic could be carried out with it. The expert of the judicial appointment had the mandate to address the defendant to demand this delivery and she did not do so and had sufficient information with the designation that it was ATPU-1 75 A polyurethane, as well as the indication of who its manufacturer was, to have located it and have carried out the appropriate tests and did not do them either, leaving open the uncertainty of whether it met the questioned characteristic. And the point is not that the procedure of the judicially appointed expert allows conclusions to be questioned, particularly when the judge is offered other evidentiary elements, with those already mentioned, that provide objective support to the doubts regarding the credibility of the expert report, issued by the judicial appointment expert.

At this point, on December 4, 2018, the Court suspended the proceedings of the process for patent infringement and claim for damages. IPH contacted MSD to try to reach an agreement. However, the agreement never came to be, and the procedure expired due to its inactivity for two years (December 2020) under Spanish regulations.^22^

Once the precautionary measures and the main procedure have been completed in this way, the damages derived from these precautionary measures still must be determined, since the fact of having obtained a final dismissal resolution means that MSD had to be liable for the possible damages caused to IFH for the adoption of said measures. In the execution lawsuit, damages caused are claimed not only by the subsidiaries of Insud Pharma, Laboratorios León Farma, S.A, in charge of manufacturing the LF Ring, and Exeltis Healthcare, S.L., in charge of its marketing in Spain, but also by all other subsidiaries of the group in charge of the distribution of the ring in other European countries such as Sweden, Norway, Finland, Germany, Belgium, Czech Republic, Slovakia, Hungary, and Poland. Several million euros were requested for this.

### Pre-Constituted Evidence in Other Countries

MSD undertook processes in other countries to try to stop the marketing of rings similar to Ornibel, which were marketed under different brands in these countries. Legal actions were taken in Germany in July 2017 and in Poland, Finland, Sweden, Norway, and Belgium in the third quarter of 2017.

In the case of Germany, MSD sent a letter to Exeltis Germany urging the stoppage of marketing of the GinoRing ring on July 27, 2017, which was responded to the following day with Exeltis’s denial of infringement. In the rest of the countries, precautionary measures were filed before obtaining them in Spain. None of the processes were successful with the requests for precautionary measures filed by MSD against the Insud Pharma ring in different European jurisdictions (Belgium, Germany, Poland, Finland, Sweden, and Norway), in which they were not adopted.

## The Strategy

The strategy is divided into three stages. As a first step to understanding potential competitors once the patent period ends, patent holders get to know the patent landscape and characteristics of the product. Once it is known, relations are broken, and a process of intense surveillance and a new business strategy begins. This new commercial strategy intensified in 2017, complemented by another consisting of trying to paralyze the entry of the competitor’s product into the market. This entails different phases that are simultaneous with the previous ones and with each other: (1) extrajudicial requests and attempts at rapprochement, (2) verification procedures to obtain information, (3) precautionary measures to justify expert reports, and (4) demand for patent infringement and claim for damages in order to maintain the action.

As recognized by the sanctioning resolution of the Spanish agency, some confidential documents collected during the inspection at the MSD headquarters that internally assessed “the action taken against Insud Pharma” were included. In other words, it was a consciously prepared strategy.

### Extrajudicial Requests and Attempts at Rapprochement

The June 13, 2017 out-of-court complaint sent by MSD to Insud Pharma ended with the following:

… in the unlikely event that your answer is negative, we hereby require that within seven days you deliver to us five copies of the authorized vaginal rings, a written response to the questions and the documentary information that is included in the annex to this letter…. We warn you that if Chemo, Exeltis or León Farma do not respond or respond negatively or evasive to this request, our clients will understand that the Kevilmare and Chemo group will begin the marketing of their vaginal rings before April 9, 2018 and that their vaginal rings will infringe the patents of MSD BV and MSD Spain, so that our clients will take the relevant legal measures to protect their rights.

Despite this requirement and the terms used, on August 8, 2017, MSD emailed Insud Pharma to find out if it would be interested in commenting on the development of its contraceptive ring and the possibility of finding a friendly way to proceed.

The initial approach assumes that they are marketing a product that infringes on their patent if they do not respond.

The willingness to talk about IPH was constant from the first moment; IPH sent a response to the first email and several subsequent ones that were not answered. The objective seemed to be not to answer but to prepare the way for the next objective: to obtain an expert report derived from judicial proceedings that could justify a judicial stoppage of production. It was already known that there would be no agreement since, in 2013, it had already been proven that the product was unlikely to infringe the original patent.

### Verification Procedures to Obtain Information

The possibility of these proceedings is justified by the impossibility of obtaining evidence demonstrating an infringement of intellectual property, which is usually in the infringer’s hands. That is why international and national regulations allow the original patent holder to go to the judge to secure evidence that can be used in a subsequent procedure that denounces the infringement. The person entitled to exercise the actions derived from the patent may ask the judge to urgently agree to the practice of proceedings to verify facts that may constitute a violation of the exclusive right granted by the patent. The idea is that they are carried out with the intervention of experts, which is the second step in the strategy.

The opposing party’s refusal to deliver certain documentation is more beneficial since the expert would have the applicant’s report (with whom the applicant met on several occasions in this instance) as basic information, and the request is said not to have been answered.

The judicial expert issued her report on September 13, 2017, in which she begins (as quoted in the issued decision) by saying what the purpose of the Expert Report is: “to analyze whether there are indications as to whether patent EP876816 (ES 2171283) has been infringed or if, on the contrary, totally rules out the occurrence of such an infraction.” The request for verification measures already requested that the analysis be nothing more than documentary, although it could have requested experimental evidence. During the interrogation, the expert said that it was not possible to perform the saturation test because she had gone to the company that manufactured the polyurethane used to manufacture the IPH ring, and it had not been given to her.^23^ Nor did she address the defendant. The conclusion reached by the expert is the following:

In view of the documents found and reviewed, it is concluded that the Ornibel vaginal ring literally infringes claim 1 of the Spanish patent ES 2171283 published on 09/01/2002, except with regard to the supersaturation of the progestogenic compound in the polymer which constitutes the core of the ring, an aspect on which they have not provided the requested information to allow the performance of the conclusive test on whether the compound is dissolved and not supersaturated, and therefore there is a possibility that the Ornibel vaginal ring infringes the ES patent. 2171283.

Therefore, the objective had been achieved. The judge specified this expert report, which served as the basis for the request for precautionary measures to suspend the competing company’s manufacturing of the ring.

### Precautionary Measures to Justify Expert Reports

The next step was to ensure that precautionary measures were adopted. The precautionary measures were requested on September 11, 2017, and another expert report was provided. They were requested without the official expert’s report having been presented, but only two days before. This is not usual, but at the same time as the verification procedures and the expert report, MSD commissioned another report from other experts who concluded the following:

The chemical experts who have prepared the expert report provided by this party together with this document requesting precautionary measures, ABG Patentes, have concluded without any doubt that ORNIBEL® and ETONOGESTREL/ETINILESTRADIOL LEON FARMA® fall within the scope of protection of the patent of my principals, so that its manufacture, export and marketing is a literal and direct infringement.

The above led to the adoption of precautionary measures on September 18, 2017, consisting of the stoppage of manufacturing and distribution. Until the marketing authorization of Ornibel in July 2017, NuvaRing was the only contraceptive ring marketed in Spain, so MSD held a 100% market share from 2002 to 2017. With the exercise of legal actions in June 2017, and, at least until the lifting of precautionary measures in December 2017, the MSD share remained close to 100%. It was above 90% until June 2018, beginning a progressive decline without falling below 60% until January 2020. In other words, a monopoly situation was maintained, but market share was diminishing.

However, the maintenance of precautionary measures depends on filing a main claim within 20 days, and it is impossible to maintain them if there is no such claim. That is why the next step was taken.

### Lawsuit for Patent Infringement and Claim for Damages in Order to Maintain the Action

The filing of a claim before the courts is a necessary consequence of the prior filing of injunctive relief. If precautionary measures are filed, the applicant for these measures must file a main claim within 30 days after they are granted, because failure to file them will render them ineffective. However, when the objective has been achieved (for example, paralyzing the commercialization of the product) with the precautionary measures, it will not be necessary for the applicant of the measures to file the claim within the legally determined term. This is because the objective of paralyzing the marketing has already been achieved and its reactivation will be more difficult or will take months or years. It is a matter of measuring the cost derived from it in comparison with the gains (benefits) that may be produced in favor of the infringer. In other words, it is a holdup/holdout problem.

Although in the specific case we are dealing with, the main proceeding was initiated within the 20 days following the adoption of the precautionary measures, the fact is that the subsequent revocation of the precautionary measures, after being appealed, had already produced certain effects. This led the affected party to accept a request for the suspension of the main proceedings in order to try to reach an agreement. The fact is that here, as we can see, the lawsuit was filed by the infringing party against the injured party and thus the precautionary measures continued, despite the suspension of the proceedings derived from the main lawsuit. In conclusion, precautionary measures are filed and obtained. The main lawsuit has been filed. It is proposed by the plaintiff (infringer) to the defendant (injured party) that the suspension of the main proceeding be granted. The suspension is requested and granted by the courts. And finally, the precautionary measures would be maintained until the main proceeding expires because it is not reactivated and resolved. The defendant (injured) would already be compensated using the precautionary measures procedure.

## Offending Conduct and Effective Judicial Protection

The exercise of actions before the Courts is not an anticompetitive practice in itself, given that it involves the right to effective judicial protection.^24^ Therefore, for this type of conduct to be classified as practices that violate competition laws, they must entail something more than an exercise of legitimate rights.

In the case under study, two different descriptions were given to these behaviors. On the one hand, the investigating body (Competition Directorate) classified them according to the following: “practices consisting of an abuse of a dominant position through a strategy of anticompetitive judicial actions aimed at delaying and hindering the entry into the market of products manufactured by Insud Pharma from at least, March 7, 2013, to April 9, 2018.”^25^ But finally, it was changed to the following: “a single infraction of abuse of dominant position — through the exercise of unfounded judicial actions with the purpose of harassing a competitor — which begins with the presentation of the request for the fact-finding procedure on June 27, 2017, and ends with the termination of the judicial procedure due to expiration on December 4, 2020.”^26^ The difference between both, in addition to the time, is that it is considered a single infraction, and the objective or intention in the first case spoke of “delaying and hindering” while in the second, it speaks of “harassing.”

### Single Violation

The Agency classified the conduct as a single and continuous violation of abuse of a dominant position by carrying out a strategy of anticompetitive judicial actions aimed at delaying and hindering the entry into the market of products manufactured by Insud Pharma. This conduct would begin when the verification procedures are commenced, since these are measures that seek the preparation of a subsequent lawsuit. The final date (dies ad quem) will be set on the date on which the infringer’s patent expires, which obviously focuses on elements that, in our opinion, are doubtful since the conduct may continue with respect to the product or service of the contrary even if there is no monopoly on the patent.

### Illegality Test: The Right to Litigate as Infringing Conduct

#### The Qualification of the Facts

The change in the classification of the facts that occurs from the investigation to the classification of the matter by the Competition Authority essentially starts from the possibility that the judicial actions constitute an anticompetitive offense, which was already analyzed in the Decisions of the European Commission of 29 July 1987,^27^ December 21, 1988,^28^ and May 21, 1996, *ITT Promedia* ^29^. It is in the latter where it is stated that to consider that the exercise of the right to litigate may constitute an anticompetitive offense in terms of an abuse of a dominant position, two cumulative circumstances with a restrictive interpretation^30^ must occur:

1. A company that has a dominant position takes legal actions that cannot reasonably be considered to be aimed at enforcing its rights and that, therefore, can only serve to harass the opposing party and,
2. That they are conceived within the framework of a plan whose purpose is to suppress competition.

It is therefore assumed that “[T]he possibility of enforcing one’s rights through judicial means and the jurisdictional control that this implies is the expression of a general principle of law that is basic in the constitutional traditions common to the European Union Member States and that was also enshrined in article 6 of the European Convention for the Protection of Human Rights and Fundamental Freedoms.” Therefore, it continues to affirm that only in exceptional circumstances could the exercise of judicial action constitute an abuse of a dominant position: a dominant position, a preconceived plan, and an objective of affecting competition.

The breakdown of normality will therefore occur, according to the Spanish resolution studied, when we encounter an unwise use of procedural resources. This unwise use must be interpreted in the context of the protection of intellectual property and the monopoly right granted by a patent,^31^ which means that whoever has it must act in accordance with the following:

- In the case of fact-checking procedures, a responsible use for this remedy means that the patent owner has done everything possible to rule out the existence of infringement by his own means and that they are necessary. In the specific case, the defendant did not refuse to offer information, although MSD denied it, and it was later proven that this was not the case. Even what was requested, which was documentation, was not enough to know whether or not the patent had been infringed, so what was requested was innocuous for the purpose of the procedure.
- In the case of precautionary measures, the application of the first element of this test means that their purpose is to guarantee the effectiveness of the future remedies on the merits, preventing the passage of time inherent in the duration of a judicial process from frustrating its effectiveness. The responsible use of precautionary measures is contrary to them “being sought by themselves” but rather depending on the purpose of the main procedure. In this specific case, they did not even wait for the expert report derived from the verification procedures.^32^

#### The Administrative Neutralization of the Facts

When the infringer alleged, in its defense, that the jurisprudence of the *AstraZeneca* case was not directly applicable to the case since it did not specifically deal with judicial actions, but rather with misleading statements to patent offices and recommended that it was the *Agria Polska* Judgment, (Case C. 373/ 17 P)^33^ which should be applied because of the jurisprudence of the Court of Justice of the European Union (CJEU), the resolution of the Spanish agency eloquently stated that the administrative body and the judicial body are different. Although in the first one, numerous complaints can be filed without this making it abusive due to the administration’s ability to archive, the judicial bodies are obliged to resolve without being able to initially neutralize these type of actions or avoid harm, also initially, that this may cause.

The fact that the judicial body granted the request for precautionary measures does not detract from the conclusion that the judicial action was intended to harass a competitor, according to General Court of the EU in *AstraZeneca*:

Thus, the success of a practice of excluding competitors by setting up barriers to entry of a regulatory nature through unlawfully obtaining exclusive rights necessarily depends on the reaction of public authorities, or even that of national courts if proceedings have been brought by competitors in order to have those rights invalidated. None the less, representations designed to obtain exclusive rights unlawfully constitute an abuse only if it is established that, in view of the objective context in which they are made, those representations are actually liable to lead the public authorities to grant the exclusive right applied for.”^34^

### Anticompetitive Purpose

The resolution states that although the abuse of a dominant position is an objective concept, this does not prevent it from considering intentionality when it is proven that the dominant operator had precisely the purpose of hindering competition.^35^ In accordance with consolidated jurisprudence of the EU,^36^ the assessment of such conduct does not require the demonstration that the practice carried out by the company in question has caused a real effect on the market; it is sufficient to demonstrate that this effect is possible or potential.^37^ The *AstraZeneca* ^38^ and *Servier* ^39^ rulings established that the intention of the dominant company plays a determining role in the court’s considerations.^40^

Following the discussion above on the exercise of legal actions, MSD had perfectly identified the competing product and its characteristics. Both on the dates contemporaneous with the exercise of these actions and afterwards, MSD assessed the success of the action to block its competitor, going so far as to accept the suspension and expiration of the main procedure as a solution to this intentional strategy.

## Objective Achieved?

As is derived from the expert report finally provided to the damage claim, MSD maintained throughout the entire time — around 6–7 months — a monopolistic market share that was maintained with the precautionary measures (preliminary injunction), allowing it to once again reach and maintain market shares close to 100% until March 2018, and face the subsequent stage of loss of market share from a much more advantageous position. The aforementioned expert report stipulates that it appears in MSD’s own annual accounts that NuvaRing’s sales suffered a decline only from 2019, due to the entry of competition into the European market, because of the entry of generics. The said decrease could have occurred earlier if precautionary measures had not been established.

The affected company chose not to invoke antitrust legislation against those using legal instruments against it primarily because of the significant costs and potential length of such proceedings. Competition litigation can often span several years, imposing a substantial financial and operational burden on parties already in a weakened position.

This is not merely about recognizing the positive role of antitrust law in fostering competitive markets, but also about preventing such legislation from being misused as a mechanism that, instead of remedying abuse, may cause further harm to those already subjected to unfair practices or abuse of dominant position.

In this context, the potential harm could have been even greater had a series of long and complex legal proceedings been initiated, thereby prolonging an adverse situation for all parties involved. However, the intervention of competition authorities serves as a balancing force, as their involvement helps to mitigate the additional burden that would otherwise fall entirely on the affected economic actors.^41^
